# HDL cholesterol efflux capacity is inversely associated with subclinical cardiovascular risk markers in young adults: The cardiovascular risk in Young Finns study

**DOI:** 10.1038/s41598-020-76146-7

**Published:** 2020-11-05

**Authors:** Monika Hunjadi, Claudia Lamina, Patrick Kahler, Tamara Bernscherer, Jorma Viikari, Terho Lehtimäki, Mika Kähönen, Mikko Hurme, Markus Juonala, Leena Taittonen, Tomi Laitinen, Eero Jokinen, Päivi Tossavainen, Nina Hutri-Kähönen, Olli Raitakari, Andreas Ritsch

**Affiliations:** 1 Department of Internal Medicine I, Medical University of Innsbruck, Anichstraße 35, 6020 Innsbruck, Austria; 2 Division of Genetic Epidemiology, Medical University of Innsbruck, Innsbruck, Austria; 3 Research Centre of Applied and Preventive Cardiovascular Medicine, University of Turku, Turku, Finland; 4 Department of Medicine, University of Turku and Division of Medicine, Turku University Hospital, Turku, Finland; 5 Department of Clinical Chemistry, Fimlab Laboratories and Finnish Cardiovascular Research Center-Tampere Faculty of Medicine and Health Technology, Tampere University, Tampere, Finland; 6 Department of Clinical Physiology, Tampere University Hospital and Faculty of Medicine and Health Technology, Tampere University, Tampere, Finland; 7 Department of Microbiology and Immunology, Faculty of Medicine and Health Technology, Tampere University and Pirkanmaa Hospital District, Tampere, Finland; 8 Department of Medicine, University of Turku and Division of Medicine, Turku University Hospital, Turku, Finland; 9 Vaasa Central Hospital, Vaasa, Finland; 10 Department of Clinical Physiology and Nuclear Medicine, Kuopio, University Hospital and University of Eastern Finland, Kuopio, Finland; 11 Department of Pediatric Cardiology, Hospital for Children and Adolescents, University of Helsinki, Helsinki, Finland; 12 Department of Pediatrics, Oulu University Hospital, PEDEGO Research Unit and MRC Oulu, University of Oulu, Oulu, Finland

**Keywords:** Molecular biology, Cardiology, Medical research, Molecular medicine, Risk factors

## Abstract

The atherogenic process begins already in childhood and progresses to symptomatic condition with age. We investigated the association of cholesterol efflux capacity (CEC) and vascular markers of subclinical atherosclerosis in healthy, young adults. CEC was determined in 2282 participants of the Young Finns study using cAMP treated ^3^H-cholesterol-labeled J774 cells. The CEC was correlated to baseline and 6-year follow-up data of cardiovascular risk factors and ultrasound measurements of arterial structure and function. CEC was higher in women, correlated with total cholesterol, HDL-C, and apolipoprotein A-I, but not with LDL-C or apolipoprotein B. Compared to the lowest CEC quartile, the highest CEC quartile was significantly associated with high CRP levels and inversely associated with adiponectin. At baseline, high CEC was associated with decreased flow-mediated dilation (FMD) and carotid artery distensibility, as well as an increased Young's modulus of elasticity, indicating adverse changes in arterial structure, and function. The association reversed with follow-up FMD data, indicating the interaction of preclinical parameters over time. A higher CEC was directly associated with a lower risk of subclinical atherosclerosis at follow-up. In young and healthy subjects, CEC was associated with important lipid risk parameters at baseline, as in older patients and CAD patients, but inversely with early risk markers for subclinical atherosclerosis.

## Introduction

Atherosclerosis is characterized by slow development from asymptomatic vascular changes beginning early in life to more advanced and symptomatic lesions with advancing age^1^. Accumulation of cholesterol into the intima layer of large arteries is a hallmark of atherosclerosis which is a dominant cause of cardiovascular disease (CVD). CVD is the most common cause of death in western societies. Low levels of high-density lipoprotein cholesterol (HDL-C) have been well established as a robust independent risk marker for CVD in the general population ^2,3^. HDL particles possess potentially antiatherogenic properties including antioxidative, anti-inflammatory, anti-apoptotic, and antithrombotic activities, they enhance endothelial function, promote endothelial repair, increase angiogenesis, suppress the production and mobilization of monocytes and neutrophils from the bone marrow, and have anti-diabetic properties ^2,4,5^.

HDL particles conventionally are characterized by their cholesterol content (HDL-C), however, the association between HDL-C and mortality is not linear over the entire range of HDL-C concentrations^6^. Recent register study showed a U-shaped relationship between HDL-C and mortality ^7^ and under several disease conditions such as chronic kidney disease, diabetes mellitus, and coronary artery disease, HDL might even lose its vasoprotective properties ^8^. It has been proven difficult to successfully reduce CVD risk with drugs increasing HDL-C such as fibrates (small increases in HDL levels), niacin (poorly tolerated in many patients), or CETP inhibitors ^9,10^. These observations are pointing towards the presence of other actions of HDL not readily reflected by HDL-C. A key function of HDL is the ability to promote the efflux of unesterified cholesterol from peripheral cells within the so-called HDL mediated reverse cholesterol transport. This process is considered atheroprotective by transferring excess cholesterol from the periphery, such as the arterial wall, back to the liver where it is secreted into the bile or converted into bile acids. The initial and rate-limiting step of reverse cholesterol transport, the macrophage-specific cholesterol efflux, can be experimentally measured as cholesterol efflux capacity (CEC) of human serum.

CEC is strongly associated with cardiovascular risk and has been proven an even better predictor than HDL-C in coronary artery disease (CAD) patients ^11–14^. Currently little is known about cholesterol efflux capacity and risk prediction in initially healthy and young individuals. Therefore, the objective of this study was twofold: first to investigate the effect of known atherosclerotic risk factors and other lipoprotein parameters on CEC within healthy young individuals; and second to evaluate the effects of CEC on preclinical parameters of atherosclerosis, independently of known risk factors, in one of the largest prospective population-based study on initially healthy young individuals.

## Subjects and methods

### Study design and participants

The Cardiovascular Risk in Young Finns (YFS) is an ongoing population-based multi-center follow-up study designed to study risk factors and precursors of cardiovascular diseases and their determinants in children and adolescents ^15^. The study participants were randomly chosen from the national register. In the 2001 follow-up, 2283 of these participants aged 24–39 years (i.e. 63.5% from the original cohort) were re-examined (Supplementary Fig. 1). A serum sample was missing from this cohort, so 2282 samples were used in the present study. None of the participating subjects had prevalent chronic heart disease. At the subsequent follow-up, 2204 individuals aged 30–45 years 2007, were re-examined. In 2001 and 2007, 2265 and 1803 study subjects, respectively, underwent vascular ultrasound examination. Participants provided written informed consent in 2001 and in 2007, and the Ethics Committee of the Hospital District of Southwest Finland approved the study. Accordingly, all methods have been carried out in accordance with relevant guidelines and regulations. All blood samples were collected and analyzed in the laboratory of the Research and Development Unit of the Social Insurance Institution, (Turku, Finland) as described by Juonala et al.^16^. Blood pressure was measured with a random zero sphygmomanometer (Hawksley & Sons Ltd, Lancing, UK) while seated after 5 min rest. The average of three measurements was used in the analysis. For determination of serum lipoprotein levels venous blood samples were drawn after an overnight fast. All lipid determinations were conducted using standard methods as previously described ^16^.

### Vascular ultrasound examination as markers of subclinical atherosclerosis

Ultrasound studies were performed using Sequoia 512 ultrasound mainframes (Acuson, CA, USA) with 13.0 MHz linear array transducers. The left carotid artery was scanned following a standardized protocol. Identical scanning protocols were used in 2001 and 2007.

The assessment of carotid artery elasticity indices (the carotid artery distensibility (CDist), Young’s elastic modulus (YEM), and the stiffness index (SI)) was conducted by a method previously described^17,18^. Briefly, the best quality cardiac cycle was selected from the 5-s clip images. The common carotid diameter 10 mm from carotid bifurcation was measured from the B-mode images using ultrasonic calipers at least twice in end-diastole and end-systole, respectively. The mean of the measurements was used as the end-diastolic and end-systolic diameter. Ultrasound and concomitant brachial blood pressure measurements were used to calculate CDist, YEM, and SI using following formulas: CDist = [(Ds − Dd)/Dd]/(Ps − Pd), YEM = [(Ps − Pd) × Dd]/[(Ds − Dd)/IMT], and SI = ln(Ps/Pd)/[(Ds − Dd)/Dd], where Dd is the diastolic diameter, Ds is the systolic diameter, Ps is systolic blood pressure, and Pd is diastolic blood pressure. CDist measures the ability of the arteries to expand as a response to pulse pressure caused by cardiac contraction and relaxation^17^. Decreased CDist has been implicated as a predictor of cardiovascular diseases^19^. Increased YEM and SI are assumed to be directly correlated with cardiovascular risk markers. YEM gives an estimate of arterial stiffness that is independent of wall (intima-media) thickness^20^. SI has been developed to reduce the impact of the curvilinear pressure-stiffness relationship on arterial stiffness and is therefore considered to be relatively independent of blood pressure ^20,21^.

In the assessment of brachial flow-mediated dilation (FMD), the left brachial artery diameter was measured both at rest and after reactive hyperemia. The increased flow was induced by inflation of a pneumatic tourniquet placed around the forearm to a pressure of 250 mmHg for 4.5 min, followed by release. Three measurements of arterial diameter were performed at end-diastole at a fixed distance from an anatomic marker, first at rest and then at 40, 60, and 80 s after cuff release. The vessel diameter in the scans after reactive hyperemia was expressed both as the change in absolute diameter (FMD) and as the percentage relative to the resting scan (FMD%) ^22^. FMD can be used to study endothelial function and impaired (decreased) FMD is a predictor of cardiovascular events ^23,24^.

The intima-media thickness (IMT) was measured as previously described ^25^. Intima media thickness is a marker of structural atherosclerosis, correlates with cardiovascular risk factors, and predicts cardiovascular events^25,26^. A dichotomous score (IMTris) is combining thick IMT and/or plaque. IMTris representing increased subclinical atherosclerosis was defined by a score 0 for normal IMT and no plaque or by score 1 for bulbus plaque and/or carotid IMT ≥ 90th percentile (0.750 mm; high IMT) and/or bulbus IMT ≥ 90th percentile^27,28^.

The described measures of preclinical atherosclerosis and all adjustment variables were available for the 2001 and 2007 re-examinations. Therefore, the values from the 2001 samples are denoted as the baseline, whereas the 2007 values as a follow-up.

### Laboratory procedures

CEC was quantified in blood samples as described ^29^. Briefly, J774.1A macrophage cells (ATCC#TIB-67) were grown in DMEM supplemented with 10% fetal bovine serum. Macrophages were resuspended in culture medium with 1% FBS and seeded into a 96-well cell culture plate (50,000 cells per well) for the assay. Lipid loading was performed overnight in 150 µL/well fresh medium with 2.5 µCi/ml [^3^H]-cholesterol. The cells were washed with PBS and equilibrated by incubation for 4 h in serum-free growth media containing 0.2% (w/v) bovine serum albumin (fatty acid-free). For activating cellular cholesterol efflux transporters J774.1A were stimulated with 0.2 mM cAMP overnight to upregulate ATP-binding cassette protein A1. Subsequently, efflux medium containing 2.8% apo B – depleted serum prepared by precipitating apoB containing lipoproteins with a mix of 8.2% tungstophosphoric acid hydrate and 6.2% 1 M MgCl_2_ was added for 4 h. All steps were performed in the presence of 5 µg/ml acyl-coenzyme A-cholesterol acyltransferase inhibitor.

All serum samples were stored at − 80 °C prior to measurement. All serum samples were measured in triplicates. Liquid scintillation counting was used to quantify the efflux of radioactive cholesterol from the cells. A serum-free sample was used to determine passive diffusion of [^3^H]-cholesterol and was treated as background (negative control). Each plate contained three control samples to allow the correction of variations between assays across the plates. Relative CEC was determined as the percentage of control CEC in [%C] using the following formula: %C = [(DPM in medium containing apoB-depleted serum − mean DPM in acceptor-free medium)/(DPM in control sample − mean DPM in acceptor-free medium)] × 100. CEC measurements were only available for the baseline samples.

### Statistical analysis

The effects of cardiovascular risk factors, separated in quartiles, on CEC levels were determined using linear models using cholesterol efflux as the dependent variable and age, sex, intake of hyperlipidemic drugs, LDL-cholesterol, HDL-cholesterol, and triglycerides as independent variables. In addition, linear models were applied with all risk factors taken as continuous variables. If necessary, highly skewed continuous variables were log-transformed (see Tables 2, 3, 4).

For the second aim, the evaluation of CEC on preclinical parameters of atherosclerosis, the following analyses were performed:

Repeated measures (from the 2001 and 2007 re-examination) were available for the preclinical parameters of atherosclerosis (FMD, IMT, SI, YEM) and all relevant potential confounding variables, which were used for adjustment. Therefore, linear mixed models with random intercepts were used, accounting for repeated measures within individuals. There was considerable interaction with time for a part of the models, indicating a different effect of HDL efflux on the outcome parameters in 2001 and 2007. This approach corresponds to analyzing the change between 2001 and 2007. In addition, linear regression models were applied, which first modeled the 2001-examination (baseline) as an outcome and then, separately, the respective measurements at follow-up (2007). For each of the three approaches, three different adjustment models were applied 1. No adjustment, 2. adjusted for age, sex, LDL-C, HDL-C, triglyceride (TG), and statin use, i.e. main cardiovascular risk factors, and 3. same as model 2 plus additionally adjusted for systolic and diastolic blood pressure and BMI, i.e. risk factors for endothelial dysfunction ^19,29^. Model 2 is considered as the main model and additional sensitivity analyses were performed based on that adjustment model. E.g. nonlinear splines were created using the baseline values and model 2 adjustment to check for linearity of effects. The linear mixed model including the interaction terms cholesterol efflux x time was also performed using this adjustment model.

To further ease the interpretability of the linear mixed models with interaction, effect plots were created, showing predicted values of the outcome variables, based on model 2 and three different selected values of HDL efflux over time. To see the effect of HDL efflux over the almost entire range of values, one rather low value (5% percentile = 81%C), the median value (= 105%C) and one rather high value (95% percentile = 128%C) was used.

To evaluate the association of HDL efflux with the binary parameters, presence of plaque (Bplaque) and IMTris-score, logistic regression models were applied for both, the prevalent (2001) as well as incident events (at 2007). The incident analyses were additionally adjusted for the respective prevalent events.

All statistical tests were 2-sided; *P* < 0.05 was considered significant. The SPSS 22.0 statistical package (SPSS Inc.) and R (version 3.5.0) including packages “nlme” and “mgcv” were used.

## Results

### Study participants

Serum samples from 2282 individuals from the 2001 follow-up of the Young Finns study were available for measurement of CEC. The clinical and biochemical characteristics of the study population are presented in Table 1. The mean participant age at baseline was 31.7 years with a higher proportion of women (55%). Lipid parameters were typical for a young population as well as the low percentage of diabetic individuals, low frequency of therapies for lipid-lowering, and hypertension. The CEC was normally distributed in this population and ranged from minimum of 33.8% C to maximum of 153.6% C (Supplementary Fig. 2). Correlation matrix for all relevant lipid, anthropometric and inflammatory parameters, which are available at baseline and follow up were show in Supplementary Fig. 3.

**Table 1:** Baseline, 2001 characteristics of the Young Finn Cohort.

Variable	Frequency	Mean	SD	Median	25th -	75th percentile
Serum specimen	2282					
Age, years	2282	31.7	4.99	32	26	35
Sex (female)	1256 (55%)					
BMI [kg/m^2^]		25.1	4.4	24.4	22	27.4
Waist [cm]		84.1	12.3	82.7	74.7	91.6
TC [mg/dL]		201	38	199	175	226
HDL-C [mg/dL]		50	12	49	41	58
LDL-C [mg/dL]		131	29	129	110	149
TG [mg/dL]		119	76	97	71	142
Lp(a) [mg/L]		125	153	63	28	152
apoA-I [g/L]		1.5	0.2	1.45	1.3	1.6
apoB [g/L]		1.1	0.25	1.02	0.87	1.2
HDL-efflux [CEC %]		104.4	14.74	104.6	95.09	114
SBP [mmHg]		117	13	115	107	125
DBP [mmHg]		70.8	10.8	70	63	77
Adiponectin [µg/ml]		9.35	4.4	8.6	6.1	11.8
CRP [mg/L]		1.93	3.95	0.75	0.33	1.87
Homocysteine [µmol/L]		9.8	3.8	9.1	7.7	10.9
SAA [µg/ml]		24.1	87.7	10.2	5.76	18.18
SDMA [µmol/L]		0.59	0.1	0.59	0.53	0.65
ADMA [µmol/L]		0.6	0.08	0.6	0.55	0.65
Mean FMD [mm]		0.22	0.14	0.22	0.13	0.32
Mean FMD [%]		6.62	4.3	3.3	3.7	9.3
Max IMT [mm]		0.62	0.1	0.63	0.56	0.68
Average IMT [mm]		.58	0.09	0.57	0.51	0.64
CDist [%/10 mm Hg]		2.2	0.74	2.1	1.6	2.7
SI		5.5	2.14	5.03	4.1	6.3
YEM [mmHg × mm]		306	146	277	210	355
Type 1 diabetes	12 (0.5%)					
Type 2 diabetes	1 (0.04%)					
Lipid lowering Tx	7 (0.3%)					
Anti-hypertensive Tx	60 (3%)					
Smoking	39 (1.7%)					
Died	0					
IMTris score*	2256 (99%)					
0	1961 (87%)					
1	295 (13%)					
Bplaque score^†^	2256 (99%)					
0	2218 (98%)					
1	38 (2%)					

BMI, body mass index; TC, total cholesterol; apo, apolipoprotein; SBP, systolic blood pressure; DBP, diastolic blood pressure; CRP, C-reactive protein; FMD, flow mediated dilation; IMT, intima-media thickness; CDist, carotid artery distensibility; YEM, Young’s elastic modulus; SI, stiffness index; Tx, therapy; valid%, percentage out of available data.

*IMTris = combined thick IMT and/or plaque (0 = normal IMT, no plaque; 1 = bulbus plaque and/or carotid IMT ≥ 90 percentile and/or bulbus IMT ≥ 90percentile).

^†^Bplaque = carotid plaque or whose maxIMT were over the 95th percentile (score 0 = no; 1 = yes).

### Cholesterol efflux capacity and cardiovascular risk factors

We investigated potential associations between lifestyle clinical factors and lipoprotein parameters with CEC. We found elevated levels of CEC in women, younger participants, and participants with elevated levels of systolic blood pressure (Table 2). CEC was directly associated with total cholesterol, HDL-C, triglycerides, and apoA-I (Table 3). After further adjustment for LDL-C, HDL-C, TG, and lipid-lowering therapy, the associations of efflux with gender, total cholesterol, apoA-I, and systolic blood pressure were eliminated due to confounding (Tables 2 and 3). ApoB showed a linear inverse association only in the extended adjusted model, though.

**Table 2:** Association of CAD risk factors with CEC at baseline as the dependent variable.

		No	Efflux, % C*	Model 1			Model 2	
				Diff/β^†^	p	Efflux, % C*	Diff/β^†^	p
**Sex**								
Women		1256	104.5 (103.6–105.3)			102.9 (102.1–103.9)		
Men		1026	102.0 (101.1–102.9)	− 2.50	**0.001**	103.9 (102.9–104.07)	0.90	0.208
**Age, years**								
< 30		699	105.4 (104.3–106.5)			105.5 (104.4–106.5)		
30–35		810	103.7 (102.7–104.7)	− 1.70	**0.028**	103.8 (102.8–104.7)	− 1.70	**0.018**
> 35		773	104.4 (103.3–105.4)	− 1.00	0.179	104.2 (103.2–105.2)	− 1.30	0.078
Age, per 1 y increase				− 0.09	0.156		− 0.10	0.0.08
**BMI [kg/m**^2^]								
Q1	< 22.0	566	103.5 (102.2–104.7)			105.4 (99.7–111.0)		
Q2	22.0–24.4	566	104.5 (103.2–105.6)	0.95	0.280	106.4 (100.8–112.1)	1.11	0.204
Q3	24.5–27.4	566	105.4 (104.2–106.7)	1.98	**0.028**	107.6 (102.0–113.2)	2.21	**0.011**
Q4	> 27.4	566	103.7 (102.5–104.9)	0.23	0.795	106.7 (101.0–112.3)	1.27	0.172
BMI, per 1 unit increase				0.04	0.560		0.08	0.263
**SBP [mmHg]**								
Q1	< 107	542	103.2 (102.0–104.5)			106.4 (100.7–112.1)		
Q2	107–115	540	103.5 (102.3–104.8)	0.31	0.727	106.2 (100.5–111.9)	− 0.17	0.835
Q3	116–125	601	105.4 (104.2–106.6)	2.15	**0.015**	107.3 (101.6–112.9)	0.92	0.278
Q4	> 125	575	105.1 (103.8–106.4)	1.88	**0.0444**	106.7 (101.0–112.3)	0.32	0.722
SBP, per 1 unit increase				0.07	**0.009**		0.02	0.334
**DBP [mmHg]**								
Q1	< 63	546	103.7 (102.5–104.9)			106.8 (101.1–112.5)		
Q2	63–70	579	104.6 (103.4–105.8)	0.87	0.313	107.0 (101.3–112.7)	0.23	0.778
Q3	70–77	546	104.1 (102.8–105.3)	0.37	0.677	106.1 (100.4–111.7)	− 0.69	0.410
Q4	> 77	583	105.1 (103.8–106.3)	1.37	0.131	107.0 (101.3–112.6)	0.19	0.830
DBP, per 1 unit increase				0.05	0.074		0.02	0.573

*Estimated marginal means and 95% confidence intervals obtained in general linear models where age, LDL-C, and HDL-C were included as continuous rather than categorical covariables;

^†^Diff, difference compared with the first category of each variable. For continuous variables, β estimates for one unit increase is also given.

Model 1: adjusted for age, sex; Model 2: adjusted for age, sex, LDL-C, HDL-C, ln(TG) and lipid lowering therapy. p-value smaller than 0.05 was considered significant (marked in bold).

**Table 3:** Association of lipoprotein parameters with CEC at baseline as the dependent variable.

	Quartile	No	Efflux, % C*	Model 1			Model 2	
				Diff/β^†^	p	Efflux, % C*	Diff/β^†^	p
**TC [mg/dL]**								
Q1	< 175	529	101.3 (100.2–102.5)			105.1 (99.2–111.0)		
Q2	175–199	583	104.6 (103.5–105.8)	3.31	** < 0.001**	106.9 (101.2–112.6)	1.80	0.071
Q3	200–226	594	104.8 (103.6–109.0)	3.46	** < 0.001**	106.7 (101.1–112.4)	1.60	0.228
Q4	> 226	576	107.1 (105.8–108.4)	5.77	** < 0.001**	108.0 (102.1–114.0)	2.94	0.145
TC, per 1 unit increase				0.05	** < 0.001**		− 0.03	0.700
**LDL-C [mg/dL]**								
Q1	< 104	646	104.3 (103.1–105.4)			107.3 (101.6–112.9)		
Q2	104–124	523	104.3 (103.0–105.6)	0.04	0.968	107.0 (101.4–112.6)	− 0.31	0.706
Q3	124–147	574	103.7 (102.5–104.9)	− 0.56	0.513	106.1 (100.5–111.8)	− 1.15	0.155
Q4	> 147	507	105.0 (103.7–106.3)	0.72	0.425	106.5 (100.8–112.2)	− 0.82	0.351
LDL-C, per 1 unit increase				0.01	0.454		− 0.01	0.286
**HDL-C, [mg/dL]**								
Q1	< 41	580	98.9 (97.7–100.1)			100.1 (94.3–105.9)		
Q2	41–49	581	102.8 (101.7–104.0)	3.9	** < 0.001**	105.1 (99.4–110.8)	5.05	** < 0.001**
Q3	50–58	554	105.4 (104.2–106.6)	6.5	** < 0.001**	108.6 (102.9–114.3)	8.50	** < 0.001**
Q4	> 58	563	110.8 (109.6–112.0)	11.9	** < 0.001**	113.6 (107.9–119.4)	13.53	** < 0.001**
HDL-C, per 1 unit increase				0.40	** < 0.001**		0.44	** < 0.001**
Effective HDL [mg/dL] (model was not adjusted for HDL-C)								
Q1	< 26	569	101.8 (100.7–103.1)			104.4 (98.4–110.3)		
Q2	26–34	568	103.1 (101.9–104.3)	1.25	0.150	106.1 (100.2–112.0)	1.76	0.046
Q3	34–43	570	104.9 (103.7–106.1)	3.01	** < 0.001**	108.5 (102.6–114.5)	4.17	** < 0.001**
Q4	> 43	569	107.5 (106.3–108.7)	5.62	** < 0.001**	111.4 (105.5–117.3)	7.04	** < 0.001**
Effective HDL, per 1 unit increase**				0.09	** < 0.001**		0.11	** < 0.001**
**TG [mg/dL]**								
Q1	< 80	625	102.4 (101.2–103.5)			103.6 (97.9–109.3)		
Q2	80–97	580	104.2 (103.0–105.4)	1.80	**0.033**	106.5 (100.9–112.1)	2.88	** < 0.001**
Q3	97–142	546	105.5 (104.2–106.7)	3.09	** < 0.001**	108.0 (102.4–113.7)	4.44	** < 0.001**
Q4	> 142	531	105.5 (104.2–106.7)	3.09	** < 0.001**	109.9 (104.2–115.5)	6.26	** < 0.001**
TG, per 1 unit increase**				0.01	** < 0.001**		0.004	** < 0.001**
**Lp(a) [mg/L]**								
Q1	< 28	567	104.1 (102.9–105.3)			106.7 (101.0–112.4)		
Q2	28–63	570	104.2 (103.0–105.4)	0.11	0.903	106.3 (100.6–111.9)	− 0.41	0.62
Q3	64–152	571	104.3 (103.1–105.5)	0.22	0.801	106.9 (101.2–112.5)	0.16	0.85
Q4	> 152	569	104.7 (103.5–106.0)	0.65	0.454	107.0 (101.3–112.6)	0.29	0.73
Lp(a), per 1 unit increase**				− 0.00	0.892		− 0.00	0.933
**apoA-I [mg/dL]**								
Q1	< 51	564	99.2 (98.1–100.4)		107.5 (101.8–113.3)			
Q2	51–57	564	103.2 (102.0–104.4)	3.95	** < 0.001**	107.7 (102.0–113.3)	0.13	0.89
Q3	58–64	566	105.0 (103.8–106.1)	5.72	** < 0.001**	105.9 (100.2–111.6)	− 1.59	0.14
Q4	> 64	564	110.8 (109.6–112.0)	11.55	** < 0.001**	104.8 (98.9–110.7)	− 2.67	0.10
apoA-I, per 1 unit increase				0.48	** < 0.001**		− 0.17	0.070
**apoB [mg/dL]**								
Q1	< 34	564	104.0 (102.7–105.2)			107.9 (102.0–113.7)		
Q2	34–40	566	104.4 (103.2–105.6)	0.40	0.641	107.4 (101.8–113.1)	− 0.44	0.650
Q3	41–48	564	104.1 (102.9–105.4)	0.18	0.842	106.0 (100.4–111.7)	− 1.84	0.146
Q4	> 48	564	104.8 (103.6–106.0)	0.84	0.355	105.6 (99.7–111.5)	− 2.27	0.211
apoB, per 1 unit increase				0.02	0.511		− 0.44	** < 0.001**

*Estimated marginal means and 95% confidence intervals obtained in general linear models where age, LDL-C, and HDL-C were included as continuous rather than categorical covariables;

^†^Diff, difference compared with the first category of each variable. For continuous variables, β estimates for one unit increase is also given.

Model 1: adjusted for age, sex; Model 2: adjusted for age, sex, LDL-C, HDL-C, ln(TG) and lipid lowering therapy.

**p-values for TG/Lp(a)/effective HDL are derived from a model with log-transformed values due to their highly skewed distributions; to obtain interpretable beta estimates, these parameters were used on original scale for estimation of β. A p-value smaller than 0.05 was considered significant (marked in bold).

### Markers of inflammation

CEC was inversely associated with the adiponectin, especially with the highest adiponectin quartile. Direct association was observed between CEC and CRP, especially in the highest quartile and also with serum amyloid A (SAA) (Table 4). No associations were found for symmetric dimethylarginine (SDMA) and asymmetrical dimethylarginine (ADMA). Additionally, we found that biologically effective HDL-C calculated using a recently developed formula based on measurements of HDL-C and SAA was directly associated with CEC ^30^.

**Table 4:** Association of inflammation parameters with CEC at baseline.

	Quartile	N	Efflux, % C*	Model 1			Model 2	
				Diff/β^†^	p	Efflux, % C*	Diff/β^†^	**p**
**Adiponectin [µg/ml]**								
Q1	< 6.1	588	103.2 (101.9–104.5)			107.1 (101.5–112.7)		
Q2	6.1–8.6	572	104.8 (103.6–106.0)	1.63	0.063	107.6 (102.0–113.3)	0.53	0.530
Q3	8.7–11.8	554	104.6 (103.2–105.8)	1.41	0.120	106.5 (100.9–112.2)	− 0.56	0.527
Q4	> 11.8	562	104.9 (103.6–106.2)	1.76	0.062	105.0 (99.4–110.7)	− 2.06	**0.0323**
Adiponectin, per 1 unit increase**				0.13	**0.018**		− 0.230	**0.029**
**C-reactive protein [mg/L]**								
Q1	< 0.33	585	103.4 (102.2–104.6)			106.2 (100.6–111.8)		
Q2	0.33–0.75	563	104.2 (103.0–105.4)	0.81	0.352	107.2 (101.5–112.8)	1.00	0.257
Q3	0.76–1.87	566	103.5 (102.3–104.7)	0.09	0.914	105.8 (100.1–111.5)	0.35	0.594
Q4	> 1.87	564	106.2 (105.0–107.4)	2.78	**0.001**	108.2 (102.5–114.0)	2.12	**0.022**
C-reactive protein, per 1 unit increase**				0.06	**0.010**		− 0.038	0.215
**Homocysteine [µmol/L]**								
Q1	< 7.7	587	103.7 (102.5–105.0)			105.4 (99.8–111.1)		
Q2	7.7–9.1	556	104.4 (103.2–105.7)	0.70	0.424	106.7 (101.1–112.3)	1.26	0.130
Q3	9.2–1.9	569	105.2 (104.0–106.4)	1.44	0.107	107.6 (102.0–113.3)	2.20	**0.009**
Q4	> 1.9	565	103.9 (102.7–105.1)	0.18	0.844	106.9 (101.3–112.6)	1.49	0.084
Homocysteine, per 1 unit increase**				0.06	0.555		0.133	**0.032**
**SAA [µg/ml]**								
Q1	< 5.76	570	102.9 (101.7–104.1)			105.7 (100.0–111.4)		
Q2	5.76–10.2	573	105.1 (103.9–106.3)	2.27	**0.009**	107.3 (101.7–112.9)	1.61	**0.053**
Q3	10.2–18.2	566	103.7 (102.4–104.9)	0.81	0.359	104.9 (99.2–110.6)	− 0.81	0.340
Q4	> 18.2	567	105.6 (104.4–106.8)	2.75	**0.002**	106.9 (101.2–112.5)	1.17	0.171
SAA, per 10 units increase**				0.01	**0.016**		0.03	0.335
**SDMA [µmol/L]**								
Q1	< .53	569	105.0 (103.8–106.2)			106.7 (101.0–112.3)		
Q2	0.53–0.59	570	104.5 (103.3–105.7)	− 0.52	0.552	106.9 (101.2–112.5)	0.23	0.784
Q3	0.60–0.65	580	103.5 (102.3–104.7)	− 1.48	0.091	106.3 (100.6–111.9)	− 0.39	0.636
Q4	> 0.65	559	104.3 (103.1–105.5)	− 0.75	0.400	106.7 (101.1–112.3)	0.04	0.965
SDMA, per 1 unit increase				− 3.25	0.302		0.25	0.934
**ADMA [µmol/L]**								
Q1	< 0.55	620	104.6 (103.5–105.8)			106.2 (100.6–111.8)		
Q2	0.55–0.60	524	103.9 (102.7–105.2)	− 0.70	0.420	106.2 (100.6–111.9)	0.03	0.969
Q3	0.61–0.65	566	104.8 (103.5–106.0)	0.11	0.896	107.6 (102.0–113.3)	1.42	0.082
Q4	> 0.65	568	103.9 (102.6–105.1)	− 0.79	0.353	107.4 (101.8–113.1)	1.21	0.143
ADMA, per 1 unit increase				− 4.07	0.275		6.72	0.062

*Estimated marginal means and 95% confidence intervals obtained in general linear models where age, LDL-C, and HDL-C were included as continuous rather than categorical covariables;

^†^Diff, difference compared with the first category of each variable. For continuous variables, β estimates for one unit increase is also given.

Model 1: adjusted for age, sex; Model 2: adjusted for age, sex, LDL-C, HDL-C, ln(TG) and lipid lowering therapy.

**p-values for Adiponectin/CRP/homocysteine/SAA are derived from a model with log-transformed values due to their highly skewed distributions; to obtain interpretable beta estimates, these parameters were used on original scale for estimation of β. A p-value smaller than 0.05 was considered significant (marked in bold).

### Preclinical parameters of cardiovascular disease

**Figure 1:**
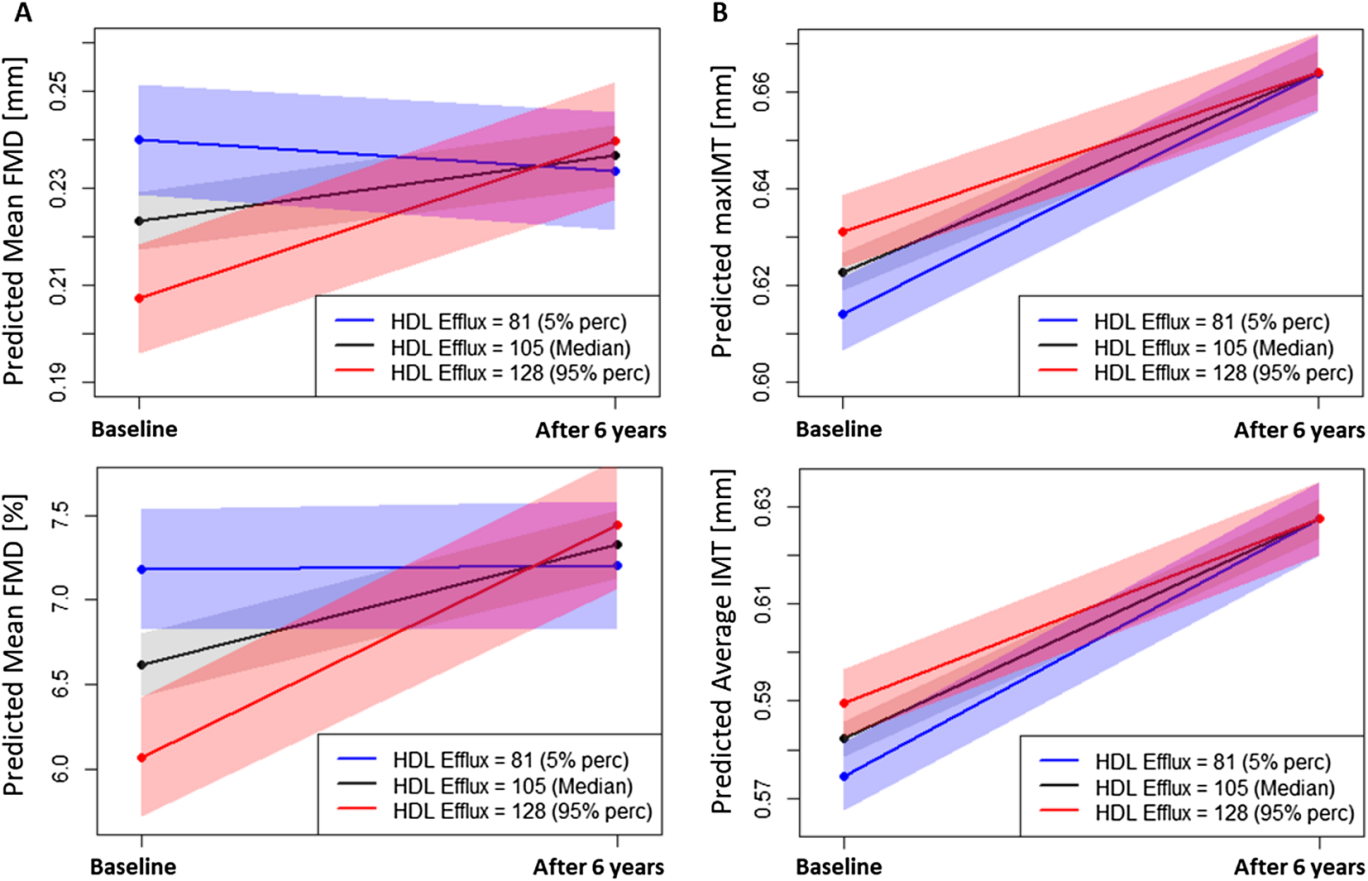
Effect plots, based on linear mixed effect models (adjusting for age, sex, LDL-C, HDL-C, TG and statin use) showing the interaction term between time and cholesterol efflux capacity on predicted values for FMD (**A**) and IMT (**B**), respectively. Very low (5% percentile, blue dots), median (black dots) and very high (95% percentile, red dots-) values are shown at two time points.

At baseline, we found an inverse correlation between CEC and flow-mediated dilation (both mean FMD of absolute and FMD%) in all adjustment models. CEC directly correlated with Young’s elastic modulus (YEM) in model 2 (Table 5). There was no indication for non-linearity in all of these linear regression models. Therefore, analyses were conducted with the continuous measurements of cholesterol efflux and beta-estimates are given for changes in 10%. The inverse correlation was not seen or even reversed regarding follow up data in 2007. Accordingly, we observed an interaction of the CEC effect with time on preclinical parameters (FMD and IMT). Participants with high CEC showed an increase in FMD over time whereas participants with low cholesterol efflux showed stable or even decreased levels of FMD (Fig. 1A). The same picture, but not as pronounced, was seen regarding intima-media thickness in these participants (Fig. 1B). For the other parameters (CDist, SI, and YEM), there was only an effect of time, but not of efflux capacity and also no interaction effect (Supplementary Fig. 4). Since the interaction of FMD depending on CEC with time was pronounced we performed a subgroup analysis dividing the population into three age groups (23/26, 29/32, and 35/38). The interaction with time is more pronounced in the lower age groups than in the older subgroup. Indicating that high CEC in the young subgroups has a more protective effect in relation to future subclinical parameter level of FMD (Supplement Fig. 5).

**Table 5:** Results of linear regression of HDL Efflux with preclinical atherosclerosis-parameters at baseline (2001) and FU (2007) and interaction p-value (HDL efflux x time) from the linear mixed model (using adjustment model 2).

	At baseline						At follow-up						Interaction with time
	Model 1^†^		Model 2^†^		Model 3^†^		Model 1^†^		Model 2^†^		Model 3^†^		
	β*	p	β*	p	β*	p	β*	p	β*	p	β*	p	p
Mean FMD	− 0.0059	**0.0045**	− 0.0075	**0.0007**	− 0.0079	**0.0003**	0.0020	0.3514	0.0022	0.3243	0.0021	0.3583	**0.0021**
Mean FMD %	− 0.1492	**0.0189**	− 0.2459	**0.0002**	− 0.2559	**0.0001**	0.1420	**0.0430**	0.0777	0.2910	0.0740	0.3120	**0.0007**
Max IMT	0.0009	0.4919	0.0026	0.0632	0.0021	0.1206	− 0.0021	0.2114	0.0002	0.8895	− 0.0001	0.9646	**0.0132**
Average IMT	0.0007	0.5861	0.0022	0.0939	0.0018	0.1643	− 0.0020	0.1941	0.0002	0.9123	− 0.0004	0.9307	**0.0159**
CDist	− 0.0062	0.5586	− 0.0132	0.2098	− 0.0081	0.4110	0.0228	**0.0393**	0.0133	0.3058	0.0132	0.2263	0.1137
SI^‡^	0.0028	0.5478	0.0058	0.2272	0.0046	0.3325	− 0.0096	0.1231	− 0.0041	0.5207	− 0.0039	0.5483	0.5630
YEM^‡^	0.0057	0.3236	0.0117	**0.0310**	0.0084	0.0920	− 0.0135	0.0672	− 0.0028	0.6903	− 0.033	0.6348	0.5333

*Per change of 10 in HDL Efflux.

^†^Model 1: unadjusted; Model 2: adjusted for age, sex, LDL-C, HDL-C, TG and statin use; Model 3: as model 2 + additionally adjusted for BMI, systolic/diastolic BP.

For the linear regression models, all adjustment variables are taken from the baseline visit.

^‡^SI and YEM were log-transformed to ensure normal distribution.

FMD, flow mediated dilation; IMT, intima-media thickness; CDist, carotid artery distensibility; YEM, Young’s elastic modulus; SI, stiffness index.

### Association of CEC with risk for high IMT and plaque at follow-up

Conventional risk factors and systemic metabolites were used to predict the 6-year incidence of high IMT (≥ 90th percentile) and/or plaque. ^19^ CEC was associated with the defined dichotomous score, representing increased subclinical atherosclerosis, the IMTris-score in 2007 in all three adjustment models (Table 6). We could deduce that the higher CEC in young and healthy participants the lower the associated IMTris score in future. There was no association with only the presence of bulbous plaque (Bplaque) or IMTris-score at baseline.

**Table 6:** Results of logistic regression of CEC with presence of plaques (Bplaque) and IMTris-score at baseline (2001) and FU (2007).

	Model 1^†^		Model 2^†^		Model 3^†^	
	β*	p	β*	p	β*	p
**At baseline**						
Bplaque (n = 38)	0.9318	0.5214	0.9403	0.6146	0.9440	0.6401
IMTris-score (n = 295)	1.0012	0.9776	1.0282	0.5551	1.0220	0.6486
**At follow-up (models are additionally adjusted for Bplaque or IMTris-Score at baseline)**						
Bplaque (n = 77)	1.1014	0.3516	1.1162	0.3355	1.1221	0.3126
IMTris-score (n = 330)	0.8584	**0.0015**	0.8983	0.0450	0.8943	**0.0380**

*Per change of 10 in CEC.

^†^Model 1: unadjusted; Model 2: adjusted for age, sex, LDL-C, HDL-C, TG and statin use; Model 3: as model 3 + additionally adjusted for BMI, systolic/diastolic BP.

All adjustment variables are taken from the baseline visit. A p-value smaller than 0.05 was considered significant (marked in bold).

Post hoc analysis in male and female subgroups showed no relevant differences (data not shown).

## Discussion

In this study, we investigated parameters influencing CEC including lipid parameters, basic clinical parameters, and classical risk factors for CVD in a large cohort of young and healthy subjects. CEC was correlated with serum total cholesterol, HDL-C, apoA-I, and TG. No relevant correlation was found between CEC and LDL-C, apoB, and Lp(a). Our results are a good indication that the assay used in this study is mainly reflecting HDL mediated CEC. Additionally, these data are consistent with earlier investigations by us and by others in elderly subjects and patients at high risk for CAD indicating that these associations may be considered valid for the majority of human subjects ^13,31^.

This view may not be applicable regarding markers of inflammation. Adiponectin is a cytokine expressed in adipose tissue. It exerts anti-inflammatory antiatherogenic properties. Adiponectin levels are decreased in obesity. It was recently proposed as a crucial modulator of HDL functionality^32^. The role of adiponectin in CVD is not clear since low levels in asymptomatic individuals have been associated with decreased CVD risk, and low level of adiponectin was shown to be a predictor of poor prognosis in patients with established CVD ^33^. While Marsche et al. showed plasma adiponectin levels to be strongly associated with efflux in healthy obese adults, we could not confirm these findings but observed an inverse association between CEC and serum adiponectin levels in our study. Plasma CRP levels, reflecting systemic inflammatory load is a validated biomarker in clinical use and in primary prevention for CVD risk prediction^34^. Similarly Marsche et al., we observed a direct association between CEC and CRP levels ^32^. CEC was previously shown to be reduced during inflammation due to microenvironmental factors, including multiple cytokines and immunocomplexes by altering the composition of the proteome and lipidome of HDL particle and inducing post-translational modifications of HDL’s protein cargo ^35–37^. Recently, we showed that SAA might modify the biological functions of HDL in several clinical settings ^30^. Serum levels of SAA were inversely associated with CEC and the newly introduced parameter effective HDL based on measurements of HDL-C corrected for SAA was strongly associated with increasing CEC. In the presented study, serum levels of SAA in the sex and age-adjusted model were directly associated with CEC. This may indicate some attenuation possibly caused by other factors than SAA in our subjects. Symmetric dimethylarginine (SDMA) and asymmetrical dimethylarginine (ADMA) have been associated with atherosclerosis and cardiovascular events in CAD patients but in our young and healthy population, this association was not evident. Taken together, in the presented study, we are not able to confirm previous investigations and even found reversed associations between cholesterol efflux capacity and markers for inflammation observed in our previous studies in CAD patients^38,39^. Accordingly, in participants of the YFS CEC was associated with adiponectin, CRP, and homocysteine only in individual quartiles. One might conclude, that these inflammatory factors may not be considered to play an early role in the development of atherosclerosis but rather be important after initiation of plaque development.

As atherosclerosis begins in childhood, it is important to recognize high-risk subjects from early risk assessments to prevent future manifestations. The majority of previous clinical studies focused on at-risk populations such as adult participants and patients undergoing angiography. HDL might lose its atheroprotective function in disease conditions as diabetes mellitus, coronary artery disease, and chronic kidney disease^8,40–42^. Most of these studies found that HDL meditated CEC is protective regarding the incidence of CVD ^11,13,14,43–45^. Several studies that examined the relationship between CVD and HDL CEC included healthy cohorts or subpopulations and found the same protective relationship. However, these studies were either relatively small or only considered middle-aged or older patients^11,14,44,46^. Subclinical parameters increased IMT, increased YEM, increased SI, but decreased CAD, and decreased FMD % are respectively markers associated with subclinical atherosclerosis and CVD. In young and healthy participants of the Young Finns Study, we report an inverse correlation between CEC and subclinical atherosclerosis regarding a marker for endothelial function, the flow-mediated dilation (FMD), and an index of arterial elasticity, the Young’s elastic modulus (YEM) at baseline. In contrast to the situation in elder individuals and CAD patients CEC seems to be inversely associated with subclinical cardiovascular risk markers in young and healthy study participants. To analyze linear regression of HDL efflux with preclinical outcomes we took into account traditional risk factors for cardiovascular disease and endothelial dysfunction. We applied three different adjustment models. We consider model 2 as the main model as this includes the main cardiovascular risk factors and also HDL-C.

If CEC and its interaction with time was associated with an outcome it was also associated after adjusting for HDL-C and other important risk factors. The associations between CEC and subclinical parameters for atherosclerosis even strengthened in these models. . This is suggesting that the association to preclinical outcome may only partly be explained by variation of confounders including HDL-C and traditional risk factors. Therefore, we could show that the capacity of HDL to promote cellular cholesterol efflux is independent of HDL-C levels and therefore one of the main drivers of the association.

Participants were followed-up after 6 years. We tested the effect of HDL mediated CEC on preclinical atherosclerosis parameters over time and found a significant interaction in FMD and IMT. The predicted FMD outcome increased over the 6 years and was highest in participants with the highest CEC. The influence of CEC on subclinical parameters may therefore increase over time and gain more significance upon age-dependent alterations in parameters associated with the development of CVD. This observation could be strengthened in subsequent subgroup analyses, where this interaction was observed only in age classes up to 32 but not in the highest tertile of participants age (Supplementary Fig. 4).

Serum CEC assay provides a useful tool in clinical research to measure changes in HDL antiatherogenic function to remove cellular cholesterol. The in vitro assay was developed to assess the cholesterol efflux capacity of HDL and was shown to be strongly inversely associated with prevalent atherosclerosis, even after adjusting for HDL-C levels^11^. CEC of HDL has been shown to be inversely associated with incident CVD ^14,44^. CEC in vitro assay is performed in a closed system missing out on the dynamic aspects of cholesterol flux through the RCT pathway, which is only possible with an in vivo RCT measurement*. *In vivo RCT measurements are performed in animal models mostly in mice. The in vivo RCT was developed by Dan Rader and colleges and involves injecting macrophages loaded with radiolabeled cholesterol into the peritoneal cavity, and measuring the appearance of the radiolabel into the plasma, liver, and feces of experimental models over time. Recently the macrophage-specific RCT method was adopted for quantification of RCT in humans ^47^. The further development of the in vivo assay would be useful in ongoing research assessing the role of key proteins regulating RCT in humans, and in evaluating the potential of novel therapeutic approaches targeting cholesterol efflux and RCT ^47^.

A limitation of our study is that the measurement of CEC does not reflect reverse cholesterol transport completely. Thus, even although we observed that CEC was related to the concentrations of HDL and its subclasses, our ex vivo model does not allow any conclusions about the contributions of the diverse subcellular mechanisms of cholesterol mobilization including ABCA1 and SR-BI in vivo. Additionally, CEC was measured once at 2001 and we were not able to adjust for possible moderate fluctuations of these parameters during the follow-up examination at 2007.

The major strengths of this work are the detailed clinical and metabolic characterization of participants of the YFS, as well as the fact that this is the first study investigating in particular the relation of CEC to preclinical parameters in a large population of initially healthy young individuals.

In summary, we could show that in one of the largest studies including young and healthy participants, CEC is inversely associated with markers of early atherosclerotic changes. This suggested that the highest CEC in this cohort is rather predicting early atherosclerotic changes. However, with progressing age this association changed to the situation mostly observed in elderly and CAD patients, where high CEC was shown to be a protective marker for CVD.

## Supplementary information

Supplementary Information.

## Abbreviations

apo: Apolipoprotein
CEC: Cholesterol efflux capacity
CVD: Cardiovascular disease
DPM: Disintegrations per unit time
HDL: High-density lipoprotein
LDL: Low-density lipoprotein
Lp(a): Lipoprotein (a)
TG: Triglycerides
FMD: Flow-mediated dilation
IMT: Intima-media thickness
CDist: Carotid artery distensibility
YEM: Young’s elastic modulus
YFS: The Cardiovascular Risk in Young Finns Study
