# Supplementary material for: HDL cholesterol efflux capacity is inversely associated with subclinical cardiovascular risk markers in young adults: The cardiovascular risk in Young Finns study
Source: Sci Rep. 2020 Nov 5;10:19223. doi: 10.1038/s41598-020-76146-7 (PMC7645719; doi:10.1038/s41598-020-76146-7)
Supplement: Supplementary file 1 — Supplementary Information. [file 41598_2020_76146_MOESM1_ESM.pdf]

HDL cholesterol efflux capacity is inversely associated with subclinical cardiovascular risk markers in young adults. The cardiovascular risk in Young Finns study

Monika Hunjadi[1*], Claudia Lamina[2], Patrick Kahler[1], Tamara Bernscherer[1], Jorma Viikari[4], Terho Lehtimäki[5], Mika Kähönen[6], Mikko Hurme[7], Markus Juonala[8], Leena Taittonen[9], Tomi Laitinen[10], Eero Jokinen[11], Päivi Tossavainen[12], Nina Hutri-Kähönen[6], Olli Raitakari[3], Andreas Ritsch[1]

[1] Department of Internal Medicine I, Medical University of Innsbruck, Innsbruck, Austria

[2] Division of Genetic Epidemiology, Medical University of Innsbruck, Innsbruck, Austria

[3] Research Centre of Applied and Preventive Cardiovascular Medicine University of Turku, Turku, Finland

[4] Department of Medicine, University of Turku and Division of Medicine, Turku University Hospital, Turku, Finland

[5] Department of Clinical Chemistry, Fimlab Laboratories and Finnish Cardiovascular Research Center-Tampere Faculty of Medicine and Health Technology, Tampere University, Finland

[6] Department of Clinical Physiology, Tampere University Hospital and Faculty of Medicine and Health Technology, Tampere University, Tampere, Finland

[7] Department of Microbiology and Immunology, Faculty of Medicine and Health Technology, Tampere University and Pirkanmaa Hospital District, Tampere, Finland

[8] Department of Medicine, University of Turku and Division of Medicine, Turku University Hospital, Turku FI

[9] Vaasa Central Hospital, Vaasa, Finland

[10] Department of Clinical Physiology and Nuclear Medicine, Kuopio, University Hospital and University of Eastern Finland, Kuopio, Finland

[11] Department of Pediatric Cardiology, Hospital for Children and Adolescents, University of Helsinki, Helsinki, Finland

[12] Department of Pediatrics, Oulu University Hospital, PEDEGO Research Unit and MRC Oulu, University of Oulu, Oulu, Finland

Correspondence: Monika Hunjadi, Department of Internal Medicine, Medical University of Innsbruck, Anichstraße 35, 6020 Innsbruck, Austria. Tel: +43 50 504 25609; Fax: +43-512-504-25608; Email: monika.hunjadi@i-med.ac.at

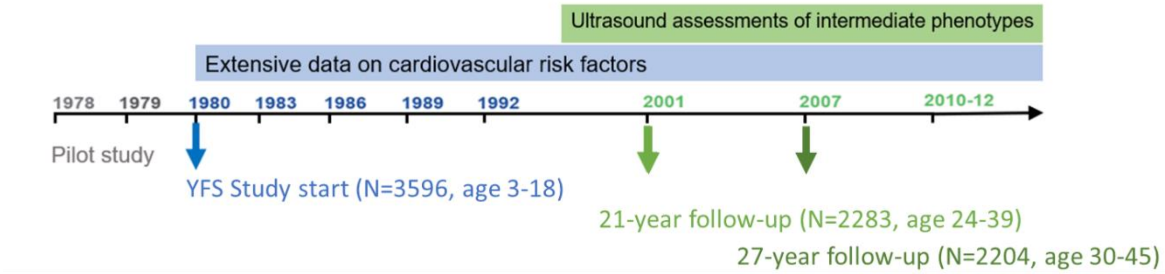

Supplementary Figure 1: The Cardiovascular Risk in Young Finns. The multi-centre study, was designed to study the risk factors and precursors of cardiovascular diseases and their determinants in children and adolescents. The first cross-sectional survey was conducted in 1980 with boys and girls in 6 age cohorts (aged 3, 6, 9, 12, 15 and 18). The subjects were randomly chosen from the national register. This cohort has been followed-up several times. Ultrasound assessment of subclinical markers for atherosclerosis was conducted for the first time in 2001 and at the follow-up in 2007.

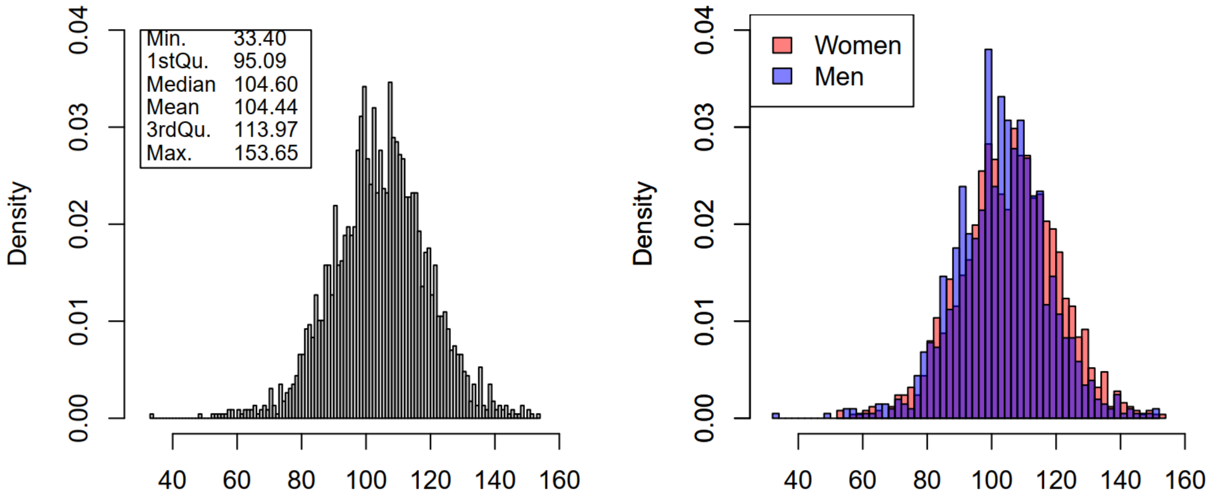

Supplementary Figure 2: Distribution of CEC in all participants with summary statistics (left panel) and stratified for men and women (right panel)

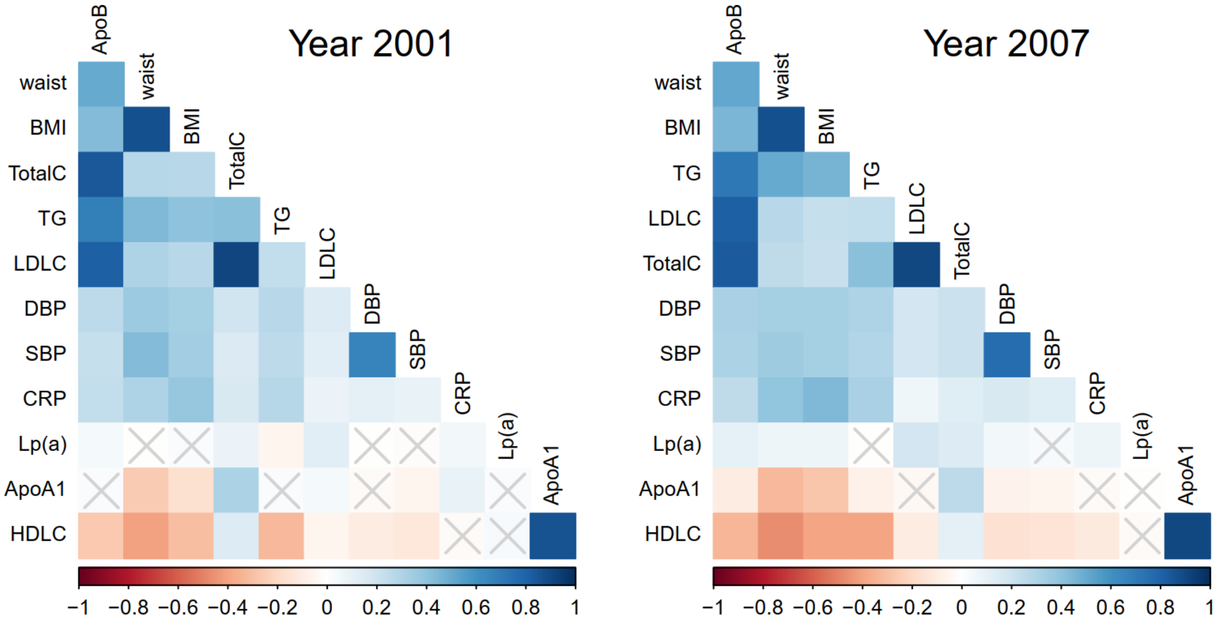

Supplementary Figure 3: Correlation matrix for all relevant lipid, anthropometric and inflammatory parameters, which are available at year 2001 and 2007. Left panel: Spearman correlation for measurements at year 2001; Right panel: Spearman correlation for measurements at year 2007. Correlation coefficients are marked in shades of red (maximum negative correlation) to blue color (maximum positive correlation). Correlation coefficients, which are not significant (p-value <0.05) are marked with a cross. Parameters are ordered by first principal components of the correlation matrix.

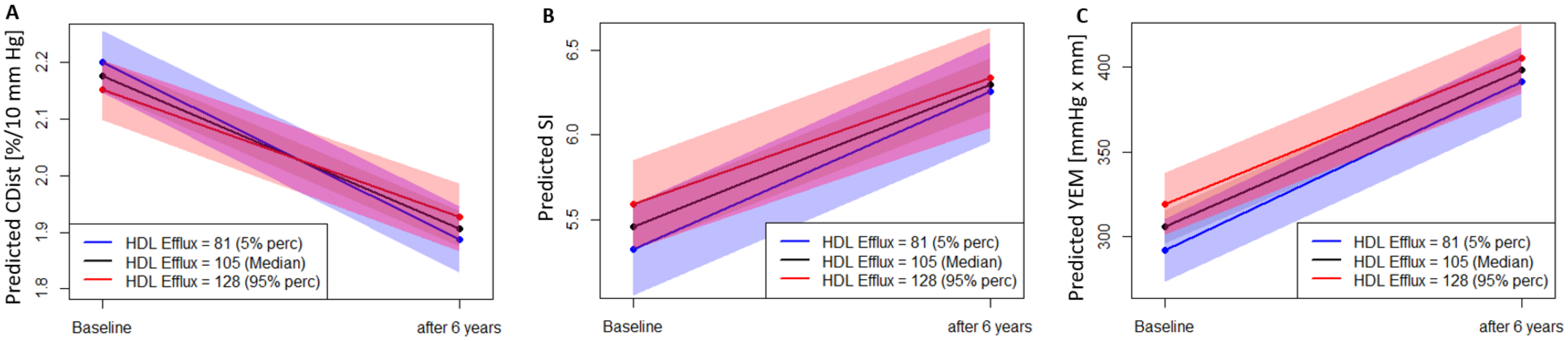

Supplementary Figure 4: Effect plots, based on linear mixed effect models (adjusting for for age, sex, LDL-C, HDL-C, TG and statin use) showing the interaction term between time and cholesterol efflux capacity on predicted values for CAC (A), SI (B) and YEM (C), respectively. Very low (5 % percentile, blue dots), median (black dots) and very high (95% percentile, red dots-) values are shown at two time points.

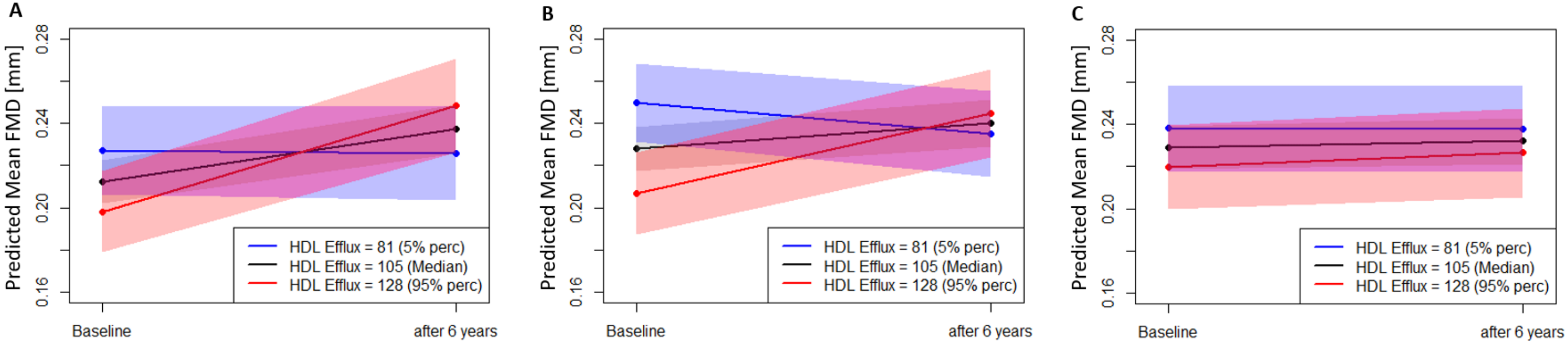

Supplementary Figure 5: Effect plots, based on linear mixed effect models (adjusting for for age, sex, LDL-C, HDL-C, TG and statin use) showing the interaction term between time and cholesterol efflux capacity on predicted values for FMD grouped for age classes 23-26 (A), 29-32 (B) and 35-38 (C), respectively. Very low (5 % percentile, blue dots), median (black dots) and very high (95% percentile, red dots-) values are shown at two time points.
